# The molecular basis of T cell receptor recognition of citrullinated tenascin-C presented by HLA-DR4

**DOI:** 10.1016/j.jbc.2025.110326

**Published:** 2025-06-02

**Authors:** Hien Thy Dao, Tiing Jen Loh, Ravi K. Sharma, Lars Klareskog, Vivianne Malmström, Hugh H. Reid, Jamie Rossjohn, Jia Jia Lim

**Affiliations:** 1 Infection and Immunity Program and Department of Biochemistry and Molecular Biology, Biomedicine Discovery Institute, Monash University, Clayton, Australia; 2 Division of Rheumatology, Department of Medicine, Karolinska Institutet, Karolinska University Hospital, Stockholm, Sweden; 3 Center for Molecular Medicine, Karolinska Institutet, Solna, Sweden; 4 Department of Clinical Immunology and Rheumatology, All India Institute of Medical Sciences, Bilaspur (H.P), India; 5 Institute of Infection and Immunity, Cardiff University School of Medicine, Heath Park, Cardiff, United Kingdom

**Keywords:** T cell receptor, rheumatoid arthritis, autoimmunity, post-translational modification, MHC

## Abstract

CD4^+^ T cell autoreactivity against citrullinated (cit) self-epitopes presented by HLA-DRB1 is associated with rheumatoid arthritis (RA) pathogenesis. We understand the molecular bases of T cell receptor (TCR) recognition of cit-fibrinogen, cit-vimentin, and cit-α-enolase epitopes, and the role of citrulline in shaping TCR repertoire usage. Nevertheless, how TCRs recognize other cit-epitopes, including tenascin-C (TNC) and how alternative citrullination positions may modulate the T cell recognition remains unclear. Here, we examined TNC^1014,1016cit^ peptide, which contains citrullination at position P-1 and P2, to study the underlying TCR-HLA-DRB1^∗^04:01-TNC^1014,1016cit^ molecular interactions. Crystal structure of HLA-DRB1^∗^04:01^TNC1014,1016cit^ at 2.4 Å resolution revealed a conserved peptide binding register to the established HLA-DRB1^∗^04:01-peptide structures, where both citrullines protruded upward. Next, we determined the crystal structure of a RA patient-derived TRAV35^+^/TRBV10-2^+^ (PB) TCR in complex with HLA-DRB1^∗^04:01^TNC1014,1016cit^ at 3.2 Å resolution. The CDR3α loop (^109^VGNTN^113^) of PB TCR formed a secondary helical conformation at the N-terminus of the peptide binding cleft, allowing extensive interactions between the P-1 and P2 citrullines of TNC^1014,1016cit^ peptide. Surface plasmon resonance, tetramer staining, and CD69 activation assays revealed that the PB TCR did not cross-react to other RA autoantigens, and the P-1-Cit, P2-Cit, and P5-Tyr of TNC^1014,1016cit^ are the key determinants underlying the strict specificity of the PB TCR. Collectively, we provide molecular insight into citrullination in modulating TCR recognition.

Rheumatoid arthritis (RA) is a T cell-mediated autoimmune disease that affects primarily the joints, and its progression leads to significant morbidity and reduced life expectancy. The disease has a prevalence of 0.5% worldwide and more than 500,000 people in Australia (1, 2). A hallmark characteristic of RA is the presence of anti-citrullinated protein antibodies (ACPA) in sera, for which >70% of the patients with RA are ACPA seropositive in established RA and before disease onset (3, 4, 5, 6, 7, 8, 9). ACPA recognizes proteins that have undergone citrullination, a post-translational modification (PTM), whereby a positively charged arginine is deiminated to the neutral amino acid citrulline, driven by peptidyl arginine deiminases (PAD), specifically PAD2 and PAD4 enzymes, leading to the emergence of neo-antigens and subsequently self-antigen immunogenicity (10, 11).

The genetic susceptibility of RA is strongly associated with the human leukocyte antigen (HLA) *loci*, specifically the HLA-DRB1 genes, which encode an HLA-DR β-chain possessing a common five-amino-acid sequence of either QKRAA, QRRAA, or RRRAA at positions 70 to 74 (12). This motif, known as the HLA-shared susceptibility epitope (SE), forms the P4 binding pocket of the HLA-DRB1 peptide binding cleft (12). With the presence of a positively charged arginine or lysine residue at position 71, a negatively charged or neutral polar residue is favored in the P4 pocket (13). This is consistent with the previous studies that the conversion of arginine into citrulline enhances the binding affinity of peptide antigen to HLA-DRB1 allomorphs bearing SE (13, 14). The prevalence and genetic susceptibility of RA can vary depending on ethnicity and racial stratification of SE-encoded *HLA-DRB1* alleles, with the *HLA-DRB1*^∗^*04*:*01* allele possessing the highest risk of RA development (odds ratio (OR) of 4.44), alongside with *HLA-DRB1*^∗^*04*:*04* (odds ratio of 4.22), *HLA-DRB1*^∗^*01*:*01* (OR of 2.17), and *HLA-DRB1*^∗^*10*:*01* (OR of 4.22) are the predominant SE-encoding alleles in Europeans (15). In Japanese and East Asians, the most common SE-encoding allele is *HLA-DRB1^∗^04*:*05* (OR of 4.22) (15, 16); Native Americans possess a common SE-coding allele of *HLA-DRB1^∗^14*:*02* (17).

The inflamed and arthritic joint synovium of RA patients is characterized by CD4^+^ T cell infiltration and the accumulation of neo-cit-epitopes originating from extracellular matrix proteins such as type II collagen (18), fibrinogen (19), tenascin-C (TNC) (20, 21), cartilage intermediate layer protein (CILP) (22), as well as cell-associated components including vimentin (22, 23) and α-enolase (24). The cit-peptides reactive CD4^+^ T cells reactive to cit-peptides derived from vimentin, α-enolase, fibrinogen, and TNC were detected in patients with RA with the HLA-DRB1^∗^04:01 (*HLA-DRA1^∗^01*:*01/HLA-DRB1^∗^04*:*01*) as well as in HLA-DRB1^∗^04:01 transgenic mice immunized with cit-peptides (23, 25, 26, 27, 28, 29). Subsequent phenotypic characterization showed an increase of cit-peptide-specific T helper 1 (Th1) effector memory CD4^+^ T cells in patients with SE^+^ RA (30, 31). Consistent with their effector role, CD4^+^ T cells produce proinflammatory cytokines in response to citrullinated antigens (21, 32), further implicating the effector function of CD4^+^ T cells in RA pathogenesis.

We have previously reported the structural basis for the mouse CD4^+^ T cell receptor (TCR) recognition of HLA-DRB1^∗^04:01 presenting cit-fibrinogen and revealed that citrullination at position P4 confers high affinity to HLA-DRB1^∗^04:01 allomorph, which in turns allows SE to co-recognize the cit-epitope and TCRs (26). In addition to P4 cit-fibrinogen, citrullination at position P2 impacted the responding TCR repertoire in immunized mice (26). Moreover, we have recently provided the key determinants underpinning TCR recognition of citrullinated vimentin and α-enolase epitopes, both of which contain P4-citrulline (27). In this study, we use a citrullinated TNC^1014,1016cit^ peptide, which contains two citrullination sites at positions P-1 and P2, to further investigate the direct impact of multi-citrullination on TCR recognition. The TRAV35^+^/TRBV10-2^+^ PB TCR was previously isolated from peripheral blood mononuclear cells (PBMC) of patient with RA (29). We show specific binding to double citrullinated TNC^1014,1016cit^ peptide presented by HLA-DRB1^∗^04:01, and the citrullines play a critical role in determining PB TCR recognition. We provide insight into the key determinants of PB TCR-HLA-DRB1^∗^04:01^TNC1014,1016cit^ recognition and the cross-reactivity of TNC^1014,1016cit^ peptide to another SE-encoded allomorphs.

## Results

### HLA-DRB1^∗^04:01^TNC1014,1016cit^ presentation

**Figure 1:**
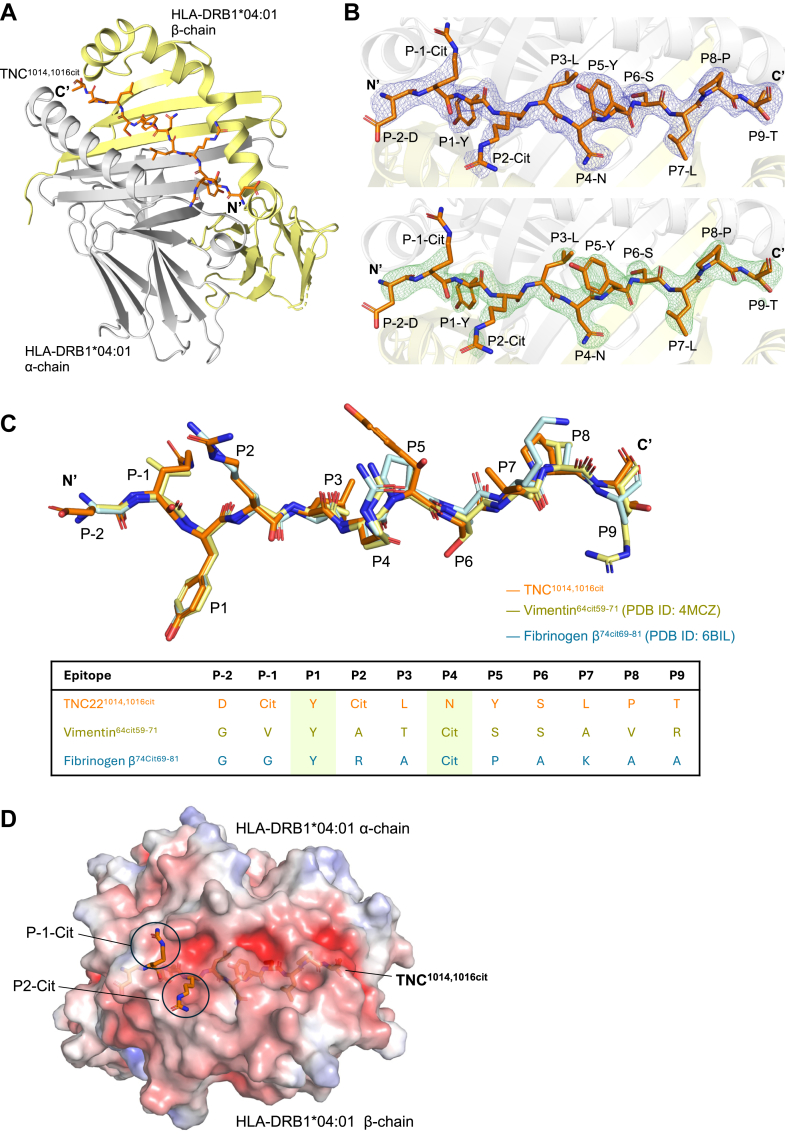
**HLA-DRB1^∗^04:01 in complex with TNC^1014,1016cit^**. *A*, cartoon representation of HLA-DRB1^∗^04:01 presenting TNC^1014,1016cit^ peptide with HLA-DRB1^∗^04:01 α- and β-chains are colored in *grey* and *yellow*, respectively, whereas TNC^1014,1016cit^ peptide is presented as *orange stick*. *B*, the refined 2mF_o_ − DF_c_ map (*top*) and SA omit map (*bottom*) of TNC^1014,1016cit^ peptide are shown in *blue* and *green*, respectively. Both maps are contoured at 1σ. *C*, overlaid structure of TNC^1014,1016cit^ (*orange*), vimentin^64cit59-71^ (*yellow*; PDB ID: 4MCZ) and fibrinogen β^74cit69-81^ peptides (*pale cyan*; PDB ID: 6BIL) from the binary complex with HLA-DRB1^∗^04:01. List of TNC^1014,1016cit^, vimentin^64cit59-71^ and fibrinogen β^74cit69-81^ peptide sequences from positions P-2 to P9. All amino acids are indicated in single-letter abbreviations, Cit = Citrulline. *D*, the Adaptive Poisson Boltzmann Solver-generated electrostatic surface of HLA-DRB1^∗^04:01^TNC1014,1016cit^ binary structure. Two citrulline residues at positions P-1 and P2 of TNC^1014,1016cit^ are circled in *black*.

To understand the register of TNC^1014,1016cit^ peptide (^1013^DcitYcitLNYSLPTG^1024^) presented by the HLA-DRB1^∗^04:01 molecule, we solved the crystal structure of HLA-DRB1^∗^04:01 presenting TNC^1014,1016cit^ (^1013^DcitYcitLNYSLPTG^1024^ KK, where two lysine residues were added at the C-terminus to improve the peptide solubility) at 2.4 Å resolution (Table 1, Fig.1*A*). The TNC^1014,1016cit^ peptide bound to HLA-DRB1^∗^04:01 in a canonical conformation, spanning from the N-terminus P-2 to C-terminus P9 residue of the peptide binding cleft, with a well-defined electron density map (Fig. 1*B*). As expected, the TNC^1014,1016cit^ peptide was aligned with the established binding motif of previously reported HLA-DRB1^∗^04:01 presenting fibrinogen β^74cit69-81^ and vimentin^64cit59-71^ peptides, where the P1 pocket accommodated by a tyrosine residue, and the P4 pocket was occupied with a neutral asparagine or citrulline residue, respectively (13, 14) (Fig. 1, *B* and *C*). In this HLA-DRB1^∗^04:01^TNC1014,1016cit^ structure, both citrullines at positions P-1 and P2 were protruding away from the binding cleft, indicating that the presence of citrulline at such positions did not impact HLA-DRB1^∗^04:01 binding (Fig. 1, *B* and *C*). This finding was supported by the similar relative binding strength reported for native and single/multiple citrullinated TNC^1013-24^ peptide bound to HLA-DRB1^∗^04:01 (EC_50_ ∼0.6–1 μM) (21). Moreover, solvent-exposed residues of the TNC^1014,1016cit^ epitope, including P5-Tyr and P8-Pro, might also play a role in interacting with the TCR (Fig. 1, *B* and *C*). The electrostatic profile of HLA-DRB1^∗^04:01^TNC1014,1016cit^ binary complex revealed a distinct neutral charged feature at P-1 and P2 of citrullinated TNC^1014,1016cit^ peptide as opposed to its native form (positive charged arginine in both position), likely suggesting the preference of the overall surface charge of the contacting TCR CDR loops which would affect the TCR specificity (Fig. 1*D*). Overall, the highly conserved binding register of HLA-DRB1^∗^04:01^TNC1014,1016cit^ binary structure suggests the potential critical role of citrullines in modulating TCR recognition.

**Table 1:** Data collection and refinement statistics of HLA-DRB1^∗^04:01^TNC1014,1016cit^, PB TCR–HLA-DRB1^∗^04:01^TNC1014,1016cit^ and PB TCR structure

Crystal structure and PDB entry	HLA-DRB1∗04:01^TNC1014,1016cit^ cit (PDB ID: 9NIH)	PB TCR-HLA-DRB1∗04:01^TNC1014,1016cit^ (PDB ID: 9NIG)	PB TCR (PDB ID: 9NII)
Data collection			
Space group	H3	P1	P22121
Cell dimensions			
*a*, *b*, *c* (Å)	119.30 119.30 73.27	76.93 88.67 126.07	109.26 119.80 176.30
*α*, *β*, *γ* (°)	90.00 90.00 120.00	81.47 76.75 65.45	90.00 90.00 90.00
Resolution (Å)	34.53–2.40 (2.49–2.40)^a^	47.39–3.20 (3.30–3.20)^a^	47.84–2.75 (2.82–2.75)^a^
R_sym_ or R_merge_	0.092 (0.352)^a^	0.112 (0.311)^a^	0.128 (1.164)^a^
CC½	0.998 (0.970)^a^	0.924 (0.506)^a^	0.996 (0.704)^a^
I/s (I)	16.4 (7.2)^a^	5 (1.3)^a^	8.3 (1.6)^a^
Completeness (%)	99.6 (99.3)^a^	98.9 (99.0)^a^	99.9 (99.9)^a^
Redundancy	10.2 (10.4)^a^	1.8 (1.8)^a^	6.1 (6.3)^a^
Refinement			
Resolution (Å)	34.53–2.40	44.320–3.2	47.84–2.75
No. reflections	15,153	48,029	60,604
R_work_/R_free_	0.1842/0.2186	0.2187/0.2604	0.2326/0.2644
No. atoms	3232	18,764	13,573
Protein	3076	18,651	13,498
Ligand/ion	46	113	8
Water	110	−	67
B-factors (Å^2^)	44.385	59.028	66.764
Protein	44.183	58.811	66.837
Ligand/ion	78.773	94.859	68.989
Water	35.682	−	51.873
R.m.s. deviations			
Bonds lengths (Å)	0.004	0.003	0.002
Bond angles (°)	0.691	0.537	0.475
Rama allowed (%)			
Rama favored (%)	97.29	96.55	95.89
Rama outlier (%)	0	0	0

a Values in parentheses refer to the highest resolution shell.

### Citrullinated TNC^1014,1016cit^ is essential for TRAV35^+^/TRBV10-2^+^ PB TCR reactivity

**Figure 2:**
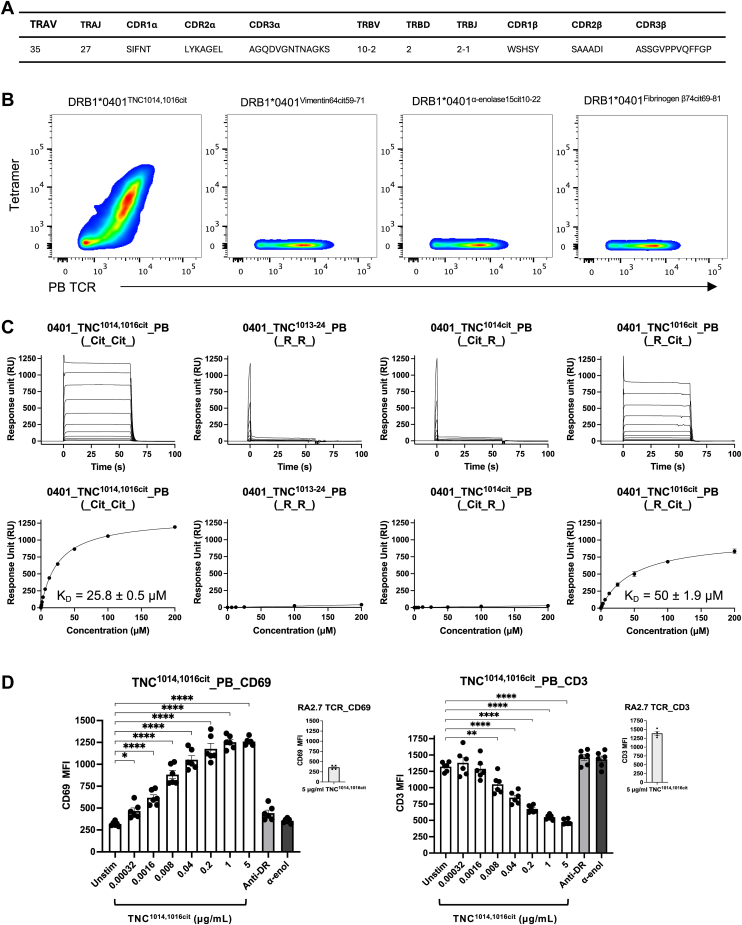
**Identification of CD4^+^ T cell restricted to HLA-DRB1^∗^04:01 presenting citrullinated TNC^1014,1016cit^ peptide**. *A*, gene segment usage and CDR loops sequence of PB TCR (29). *B*, *in vitro* PB TCR expression and tetramer staining for individual HLA-DRB1^∗^04:01 tetramers presenting citrullinated RA autoantigens, including TNC^1014,1016cit^, Vimentin^64cit59-71^, α-enolase^15cit10-22^, and Fibrinogen β^74cit69-81^ peptides. Gating strategy is shown in Fig.S1. *C*, binding affinity of PB TCR against HLA-DRB1^∗^04:01 presenting TNC^1014,1016cit^, native TNC^1013-24^, TNC^1014cit^ and TNC^1016cit^ peptides. HLA-DRB1^∗^04:01^CLIP^ was immobilized in the reference flow cell to control non-specific binding. PB TCR equilibrium affinity constants (K_D_) values were determined from three independent experiments in duplicate and curve fitted using a 1:1 binding model. For each concentration, the points represent the mean values, and the error bars correspond to ± s.e.m. from three independent experiments in *duplicate*. *D*, activation assay of PB TCR transduced SKW3 T cells against BLCL 9031 expressing HLA-DRB1^∗^04:01 stimulated with TNC^1014,1016cit^ peptide. Upregulation CD69 expression (*left*) and down-regulation CD3 expression (*right*) of PB TCR upon serial dilution of TNC^1014,1016cit^ peptide are shown in the bar chart. Three independent experiments in *duplicate* were performed. *p*-values were determined by one-way ANOVA with Dunnett’s multiple comparison testing, ∗*p* ≤ 0.05, ∗∗*p* ≤ 0.01, ∗∗∗∗*p* ≤ 0.0001, and error bars correspond to ± s.e.m. from three independent experiments in *duplicate*.

A human *TRAV35*^*+*^*/TRBV10-2*^*+*^ CD4^+^ T cell clone was previously isolated from the PBMC of a HLA-DRB1^∗^04:01 patient with ACPA-positive RA, *via* HLA-DRB1^∗^04:01-TNC^1014,1016cit^ tetramer (29) (Fig. 2*A*). To investigate the antigen specificity and impact of citrullination in TCR recognition, we transiently expressed TRAV35^+^/TRBV10-2^+^ PB TCR in the HEK293 T cell line and stained with individual HLA-DRB1^∗^04:01 tetramers presenting TNC^1014,1016cit^, vimentin^64cit59-71^, a-enolase^15cit10-22^, or fibrinogen β^74cit69-81^, respectively (Figs. 2*B* and S1). As expected, TRAV35^+^/TRBV10-2^+^ PB TCR bound specifically to TNC^1014,1016cit^ peptide and did not cross-react to other RA autoantigens (Fig. 2*B*). Next, we expressed, refolded, and purified TRAV35^+^/TRBV10-2^+^ PB TCR and subsequently determined the steady state binding affinity of this TCR with four different variants of TNC^1013-24^ peptide, namely, TNC^1014,1016cit^ (P-1 and P2 citrullinated), TNC^1014cit^ (P-1 citrullinated), TNC^1016cit^ (P2 citrullinated), and native TNC^1013-24^ peptide *via* surface plasmon resonance (SPR) (Fig. 2*C*). The affinity of PB TCR-HLA-DRB1^∗^04:01-TNC^1014,1016cit^ fell within the relative range of TCR-pMHC II interaction (33) as well as previously determined TCRs-HLA-DRB1^∗^04:01-cit-epitopes (26, 27). The PB TCR bound strongest in the presence of both citrullines to HLA-DRB1^∗^04:01^TNC^^1014,1016cit^, with a K_D_ = 25.8 μM and did not recognize native TNC^1013-24^ peptide, highlighting the essential role of citrullination in PB TCR recognition (Fig. 2*C*). In particular, TNC^1014cit^ had a critical impact on PB TCR recognition, whereas TNC^1016cit^ displayed a twofold weaker affinity (K_D_ = 50 μM) to PB TCR than the double citrullinated epitope (Fig. 2*C*).

Subsequently, we used the T cell activation assay to provide insight into the functionality of the PB TCR and HLA-DRB1^∗^04:01^TNC1014,1016cit^ interaction. Here, we generated a PB TCR transduced SKW-3 CD4^+^ T cell line and measured the expression of CD69 and CD3 cell surface markers as an indicator of T cell activation. We observed an increase in CD69 expression and a concomitant downregulation of CD3 expression, which corresponded with a dose-dependent response to the TNC^1014,1016cit^ peptide concentration, suggesting that PB T cell line activated the TCR signaling pathway in response to TNC^1014,1016cit^ peptide recognition (Fig. 2*D*). In particular, the PB TCR was highly reactive to TNC^1014,1016cit^ peptide, with approximately 0.32 ng/ml of peptide was sufficient to significantly activate PB TCR with a maximal response reached at ∼ 1 μg/ml (Fig. 2*D*). Here, we used the HLA-DRB1^∗^04:01^α-enolase15cit10-22^ restricted TCR, RA2.7 (27) and an anti-HLA-DR blocking antibody (clone LB3.1) as controls, indicating that the activation of PB TCR is citrullinated TNC^1014,1016cit^-specific and in a HLA-DRB1^∗^04:01-dependent manner (Fig. 2*D*). Taken together, double citrullination at TNC^1014,1016cit^ peptide is a key determinant underlying PB TCR recognition and reactivity, with citrulline at P2 being critical for recognition, while citrulline at P-1 enhances recognition.

### Docking topology of TRAV35^+^/TRBV10-2^+^ PB TCR on HLA-DRB1^∗^04:01^TNC1014,1016cit^

**Figure 3:**
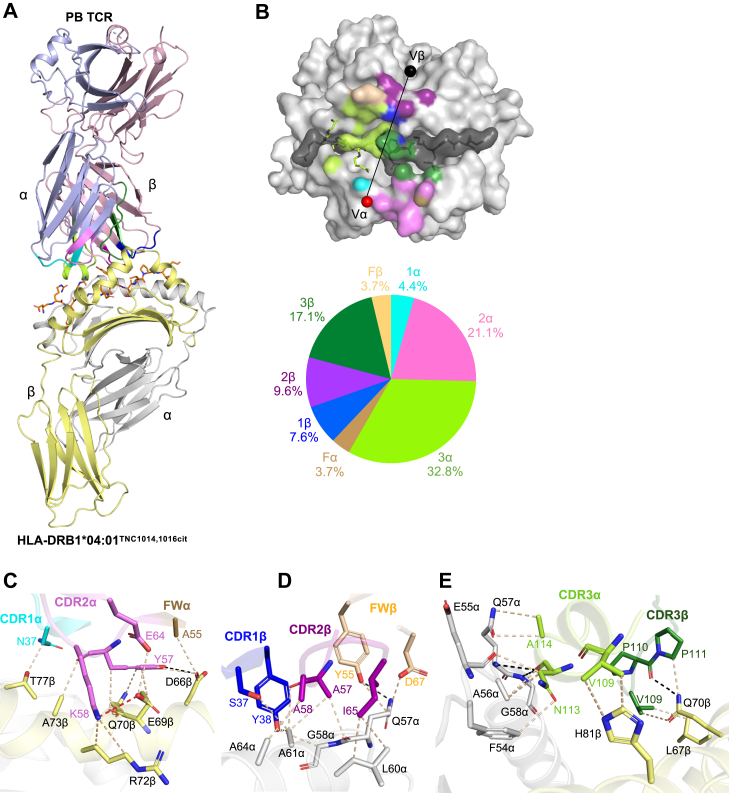
**PB TCR recognition of HLA-DRB1^∗^04:01^TNC1014,1016cit^**. *A*, overall cartoon representation of PB TCR complexed to HLA-DRB1^∗^04:01^TNC1014,1016cit^. The HLA-DRB1^∗^04:01 α- and β-chains are highlighted in *grey* and *yellow*, whereas PB TCR α- and β-chains are presented in *light purple* and *light pink*, respectively. The TNC^1014,1016cit^ peptide is presented as *orange sticks*. The CDR1α, 2α and 3α loops are colored in *cyan*, *violet* and *light green*, while the CDR1β, 2β and 3β are highlighted in *blue*, *purple* and *dark green*, respectively. The FWα residues are colored in sand and FWβ residues are presented in beige. *B*, *(top)* surface representation of PB TCR footprint on the HLA-DRB1^∗^04:01^TNC1014,1016cit^. The atoms from HLA-DRB1^∗^04:01^TNC1014,1016cit^ interacting with PB TCR are colored according to the nearest CDR loops that they are interacting with. The Vα and Vβ center of mass positions are presented as *spheres* in *red* and *black*, respectively, connecting *via* a black line. (*Bottom*) pie chart highlights the relative contribution of CDR loops to the interface of HLA-DRB1^∗^04:01^TNC1014,1016cit^. Detailed interactions of PB TCR between (*C*) germline encoded CDR1α, CDR2α and FWα, (*D*) CDR1β, CDR2β and FWβ and (*E)* non-germline encoded CDR3α and CDR3β with HLA-DRB1^∗^04:01 are shown. *Black dashes* represent H-bond within 3.5 Å, *beige dashes* correspond to VdW interaction within 4 Å and *red dashes* denote disulfide bond within 4.5 Å distance. All amino acids are indicated in single-letter abbreviations.

To understand the molecular basis underpinning the specific recognition of PB TCR for TNC^1014,1016cit^ presented by HLA-DRB1^∗^04:01, we determined the ternary complex structure at 3.2 Å resolution (Table 1, Figs. 3*A*, and S2*A*). The electron density map at the interface of the PB TCR and HLA-DRB1^∗^04:01^TNC1014,1016cit^ was clearly defined (Fig. S2*A*). The PB TCR docked canonically at an angle of ∼70° across the central region of peptide binding cleft, with a total Buried surface area of ∼1910 Å^2^ over HLA-DRB1^∗^04:01^TNC1014,1016cit^. (Fig. 3*B* and Table S1). The TRAV35^+^/TRBV10-2^+^ PB TCR chain usage was biased toward the α-chain that constituted ∼62% of BSA as compared to ∼38% of the β-chain (Fig. 3*B* and Table S1). Particularly, the PB TCR CDR3α and 2α made major contributions to the HLA-DRB1^∗^04:01^TNC1014,1016cit^ interaction, by contributing 32.8% and 21.1% of total BSA, respectively, followed by CDR1α (4.4%) and framework α (FWα) (3.7%) (Fig. 3*B* and Table S1). In contrast, CDR3β, 2β, 1β and FWβ contributed 17.1%, 9.6%, 7.6% and 3.7% of total BSA, respectively (Fig. 3*B* and Table S1). Intriguingly, the TNC^1014,1016cit^ peptide contacts were mainly derived from non-germline encoded CDR3α and 3β loops, whereas germline encoded CDR1β, 2β, 1α and 2α loops contributed to the interaction with HLA-DRB1^∗^04:01 (Fig. 3*B*).

### Molecular basis of TRAV35^+^/TRBV10-2^+^ PB TCR recognition of HLA-DRB1^∗^04:01

The germline encoded TRAV35^+^/TRBV10-2^+^ PB TCR made extensive contacts with HLA-DRB1^∗^04:01, in which CDR-α and -β loops mainly interacted with HLA-DRB1^∗^04:01 β- and α-chains, respectively, indicating a typical canonical docking mode of TCR-peptide-major histocompatibility complex II (pMHC II) (33) (Fig. 3, *C–E* and Table S2). Here, CDR1α (Asn^37^) and FWα (Ala^55^) made contacts with Thr^77^ and Asp^66^ of the HLA-DRB1^∗^04:01 β-chain, respectively, *via* van der Waals (VdW) interactions (Fig. 3*C* and Table S2). Notably, germline-encoded CDR2α residues (Tyr^57^, Lys^58^, and Glu^64^) formed multiple interactions with the DRB1^∗^04:01 β-chain, including SE residues (Gln^70^, Ala^73^, and Arg^72^) and adjacent residues (Asp^66^ and Glu^69^) *via* H-bonds, salt bridge, and VdWs, suggesting the importance of conserved *TRAV* gene usage in HLA-DRB1^∗^04:01 β-chain recognition (Fig. 3*C* and Table S2). For the PB TCR β-chain, CDR1β (Ser^37^ and Tyr^38^) and CDR2β (Ala^57^, Ala^58^, and Ile^65^) formed multiple VdWs with the HLA-DRB1^∗^04:01 α-chain (Gln^57^, Gly^58^, Leu^60^, Ala^61^, and Ala^64^) (Fig. 3*D* and Table S2). Tyr^55^ and Asp^67^ of PB TCR FWβ also interacted with Gln^57^ of HLA-DRB1^∗^04:01 α-chain (Fig. 3*D* and Table S2). In the context of non-germline encoded interface, CDR3α (Val^109^, Asn^113^, and Ala^114^) and CDR3β (Val^109^, Pro^110^, and Pro^111^) interacted with HLA-DRB1^∗^04:01 α- (Phe^54^, Glu^55^, Ala^56^, Gln^57^, and Gly^58^) and β-chains (Leu^67^, Gln^70^, and His^81^), respectively, *via* H-bonds and VdWs (Fig. 3*E* and Table S2). Overall, the substantial contribution of germline-encoded residues of TRAV35^+^/TRBV10-2^+^ PB TCR, alongside non-germline-encoded residues, highlights the potential TCR gene usage preference specific for HLA-DRB1^∗^04:01 engagement.

### Altered structural changes in the CDR3**α** loop are critical for co-recognition of PB TCR-TNC^1014,1016cit^

**Figure 4:**
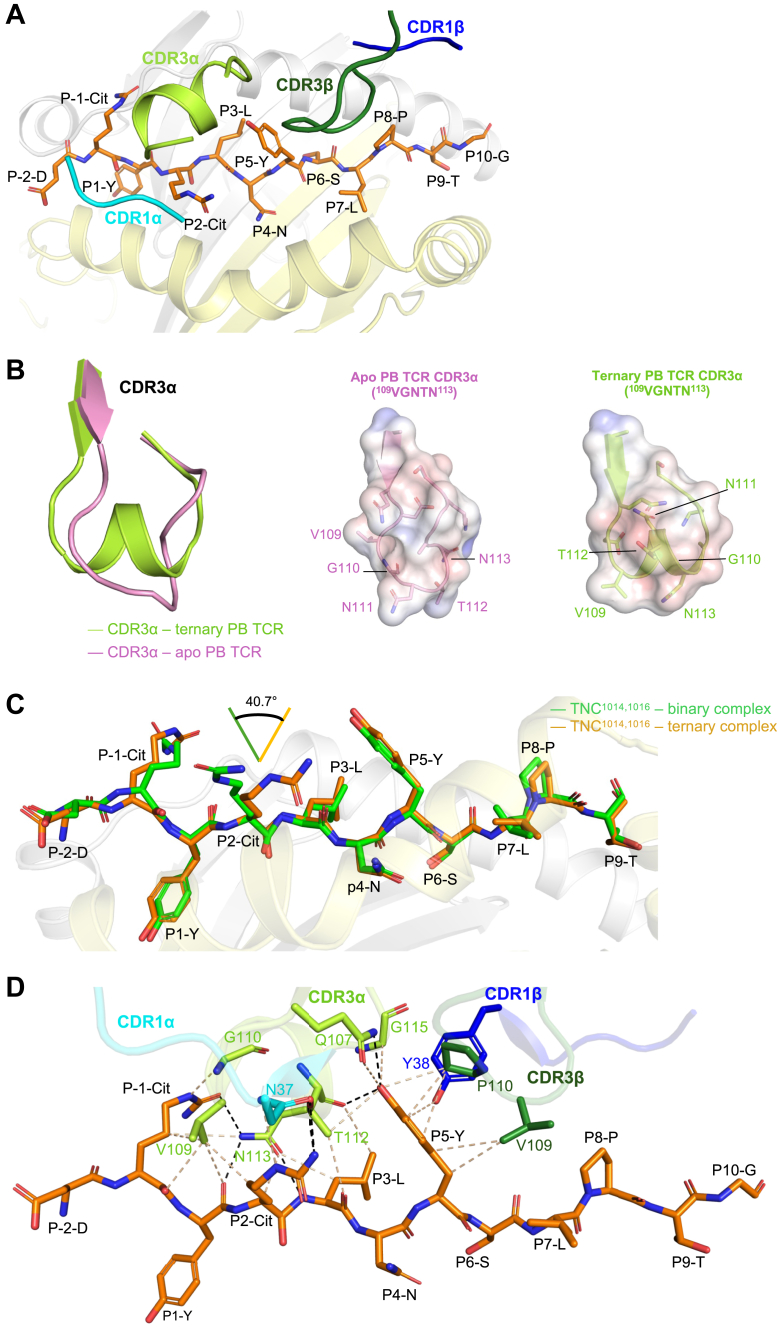
**TNC^1014,1016cit^ peptide-mediated PB TCR interactions**. *A*, schematic representation depicting the docking topology of CDR1α, 3α, 1β and 3β loops atop of HLA-DRB1^∗^04:01^TNC1014,1016cit^. HLA-DRB1^∗^04:01 α- and β-chains are colored in *grey* and *yellow*, while the CDR1α, 3α, 1β and 3β loops are highlighted in *cyan*, *light green*, *blue* and *dark green*, respectively. TNC^1014,1016cit^ peptide is presented as *orange stick*. *B*, *(left*) overlaid CDR3α loop from the unliganded PB TCR apo structure, in *pin*k, and from the PB TCR-HLA-DRB1^∗^04:01^TNC1014,1016cit^ ternary complex, in *light green*. The Adaptive Poisson Boltzmann Solver-generated electrostatic surface of (*middle*) apo PB TCR-CDR3α loop and (*right*) holo PB TCR-CDR3α loop, displaying the arrangement of ^109^VGNTN^113^ sequence. *C*, overlaid P-2 to P9 residues of TNC^1014,1016cit^ peptide from the HLA-DRB1^∗^04:01^TNC1014,1016cit^ binary complex (*green*) and from the PB TCR-HLA-DRB1^∗^04:01^TNC1014,1016cit^ ternary complex (*orange*). *D*, detailed interactions of PB TCR in contact with the TNC^1014,1016^ peptide are shown in *sticks*. *Black dashes* represent H-bond within 3.5 Å distance *and beige dashes* correspond to VdW interaction within 4 Å distance. All amino acids are indicated in single-letter abbreviations. Citrulline denotes as Cit.

The PB TCR and TNC^1014,1016cit^ peptide interactions were primarily driven by the CDR3α and CDR3β loops, followed by limited involvement of CDR1α and CDR1β (Fig. 4*A*). Five residues in CDR3α loop (^109^VGNTN^113^) formed a secondary helical conformation which in turn sat atop the N-terminus of TNC^1014,1016cit^ peptide at position P-1 to P3 pocket (Fig. 4, *A* and *B*). To understand whether this secondary structure transition of the CDR3α loop is ligand driven, we also determined the structure of the apo form of PB TCR at 2.75 Å resolution (Table 1 and Fig. S2*B*). Superposition of PB TCR-HLA-DRB1^∗^04:01^TNC^^1014,1016cit^ holo and PB TCR apo form at the Cα backbone of the TCR showed a very subtle change with a root mean square deviation (r.m.sd) value of 0.57 Å (Fig. S2*B*). In the PB TCR apo form, the CDR3α loop was unstructured, whereupon Asn^111^ and Thr^112^ were positioned downward (Fig. 4*B*). While in the PB TCR holo state, the ^109^VGNTN^113^ of the CDR3α loop reoriented with Val^109^ and Asn^113^ to point downward facing HLA-DRB1^∗^04:01^TNC^^1014,1016cit^. Thus, the altered conformational transition of the CDR3α loop in the PB TCR-TNC^1014,1016cit^ holo state is ligand driven.

In the context of the TNC^1014,1016cit^ peptide, there was a rearrangement of the citrulline residues upon CDR3α loop docking. Namely, citrulline at P2 shifted ∼40° toward the C-terminus, alongside with subtle rearrangement of the P-1 citrulline at the N-terminus, which allowed the docking of the conformationally rearranged CDR3α loop (Fig. 4*C*).

### Detailed interactions of TRAV35^+^/TRBV10-2^+^ PB TCR and TNC^1014,1016cit^ peptide

At the PB TCR-TNC^1014,1016cit^ interface, the nature of the α-helical turn in the CDR3α loop enabled extensive interactions between P-1 to P3 residues of the TNC^1014,1016cit^ peptide. Here, Val^109^ projected downward and positioned at the center of the P-1 and P2 citrullines, forming multiple contacts with both citrullines, as well as the main chain interactions with P1-Tyr (Fig. 4*D* and Table S2). The adjacent Gly^110^ and Asn^113^ of CDR3α also made extensive H-bonds and VdW contacts with P-1 citrulline, as well as main chain interactions with P1-Tyr, P2-Cit, and P3-Leu (Fig. 4*D* and Table S2). Moreover, Thr^112^ and Asn^37^ of CDR3α and CDR1α loops, respectively, contacted with P2 citrulline within the H-bond distance (Fig. 4*D* and Table S2). Another distinct feature of PB TCR recognition of TNC^1014,1016cit^ peptide was observed at Position 5, where numerous interactions were made between P5-Tyr and CDR3α (Thr^112^, Gln^107^, and Gly^115^), CDR3β (Pro^110^ and Val^109^), and CDR1β (Tyr^38^) (Fig. 4*D* and Table S2). The neutral feature of citrullinated TNC^1014,1016cit^ peptide in the presence of both P-1 and P2 citrullines, complemented with the hydrophobic Val^109^ of CDR3α, further explains the role of citrullination in recognizing PB TCR (Fig. 4*D*). Such hydrophobic feature of Val^109^ in CDR3α likely disfavored the positively charged arginine in the native TNC^1013-24^ peptide or single citrullinated TNC^1016cit^ peptide due to charge repulsion (Fig. 4*D*). The structural analyses were consistent with the SPR data of high affinity of PB TCR towards double citrullinated TNC^1014,1016cit^ peptide as opposed to P2 citrullinated TNC^1016cit^ peptide (Fig. 4*D*). Collectively, both citrullines at P-1 and P2, alongside with P5-Tyr, are the key determinants for TRAV35^+^/TRBV10-2^+^ PB TCR recognition.

### Energetic determinants underlying PB TCR recognition and HLA-DRB1^∗^04:01^TNC1014,1016cit^

**Figure 5:**
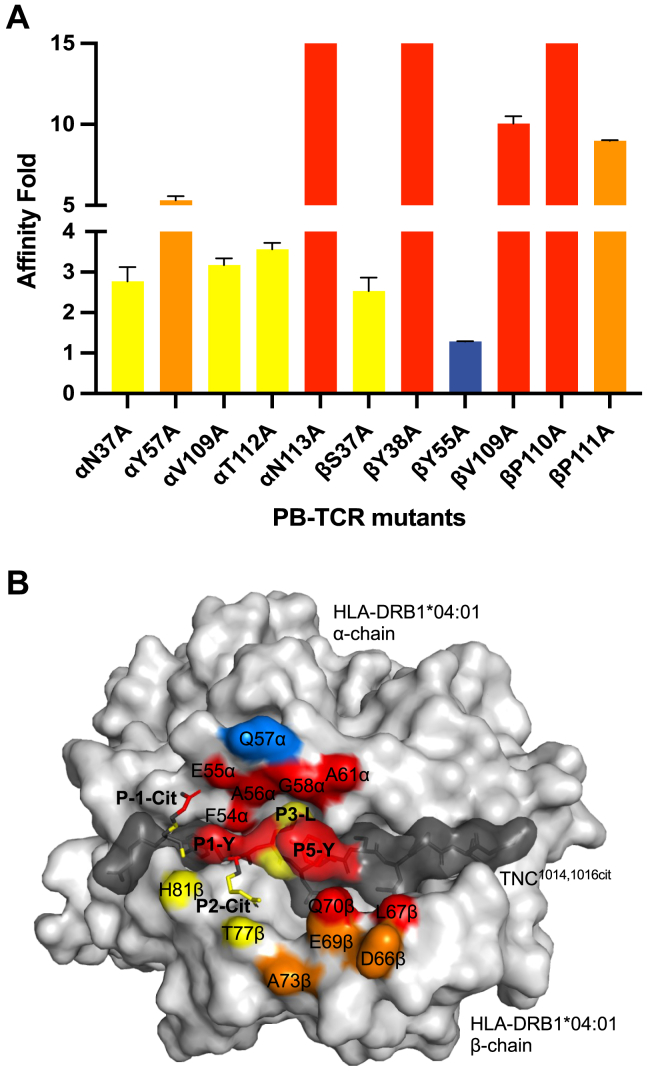
**Effect of PB TCR point mutations at the HLA-DRB1^∗^04:01^TNC1014,1016cit^ interface.***A*, the affinity in fold of PB TCR mutants as compared to the native PB TCR was calculated corresponded to equilibrium affinity constants (K_D_) values in Figure S3 and Table S3. The impact of each mutation was categorized as no effect (<2-fold reduced affinity compared to wildtype, *blue*), moderate (2-5-fold reduced affinity, *yellow*), severe (5–10-fold reduced affinity, *orange*), and deleterious (>10-fold reduced affinity, *red*). All data were derived from two independent measurements in *duplicate* and the error bars correspond to ± s.e.m. *B*, energetic footprint of PB TCR on HLA-DRB1^∗^04:01^TNC1014,1016cit^ complex. The impact of each mutation is colored according to (*A*). Surface representation of HLA-DRB1^∗^04:01 and the TNC^1014,1016cit^ peptide are colored in light grey and dark grey, respectively.

To define the energetically important residues of the TRAV35^+^/TRBV10-2^+^ PB TCR attributing to HLA-DRB1^∗^04:01^TNC1014,1016cit^ recognition, we conducted an alanine-scanning mutagenesis of a panel of 11 residues on PB TCR that are involved in contacts with HLA-DRB1^∗^04:01^TNC1014,1016cit^, and analyzed their impact on TCR-pMHC II binding using SPR. The impact of each mutation was classified into four categories: no effect (<2-fold reduced affinity compared to wildtype), moderate (2-5-fold reduced affinity), severe (5–10-fold reduced affinity), and deleterious (>10-fold reduced affinity). Alanine substitution of Asn^113^ (CDR3α), Tyr^38^ (CDR1β), Val^109^, and Pro^110^ (CDR3β) residues which co-contacted both HLA-DRB1^∗^04:01 and TNC^1014,1016cit^ peptide had a deleterious effect on PB TCR recognition (Figs. 5*A*, S3 and Table S3). CDR2α (Tyr^57^) and CDR3β (Pro^111^) which contacted HLA-DRB1^∗^04:01 particularly at the shared epitope also revealed severe impact on HLA-DRB1^∗^04:01^TNC1014,1016cit^ recognition with >5-fold reduced in affinity when compared to wildtype TCR (Figs. 5*A*, S3 and Table S3). Other residues, including CDR1α (Asn^37^), CDR3α (Val^109^ and Thr^112^), and CDR1β (Ser^37^), which interacted with either HLA-DRB1^∗^04:01 or TNC^1014,1016cit^ peptide, showed a moderate impact on PB TCR recognition. In contrast, FWβ (Tyr^55^) had no impact on pHLA recognition (Figs. 5*A*, S3 and Table S3). Collectively, the impact of germline encoded and variable residues in CDR loops formed an energetic hotspot on HLA-DRB1^∗^04:01^TNC1014,1016cit^ at the N-terminus region P1-P5 across the HLA-DRB1^∗^04:01 peptide-binding cleft (Fig. 5*B*).

### Role of HLA-DRB1^∗^04:01 SE in TCR recognition

**Figure 6:**
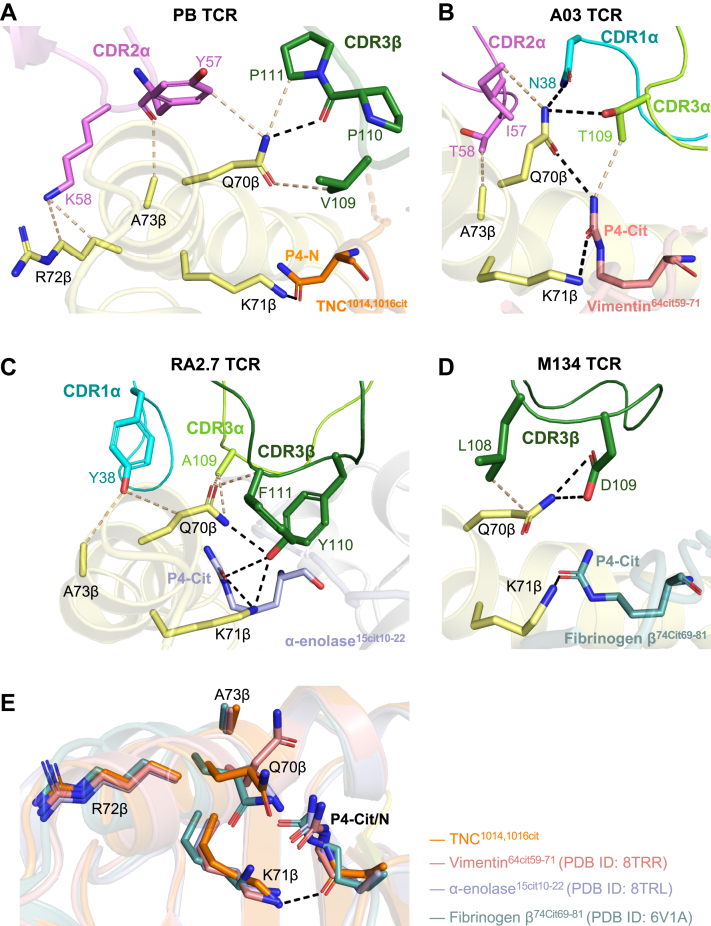
**Detailed SE interactions of HLA-DRB1^∗^04:01 with cit-epitopes and restricted TCRs.** Detailed SE interactions for (*A*) TNC^1014,1016cit^ restricted PB TCR, (*B*) vimentin^64cit59-71^ restricted A03 TCR (PDB ID: 8TRR), (*C*) α-enolase^15cit10-22^ restricted RA2.7 TCR (PDB ID: 8TRL) and (*D*) fibrinogen β^74cit69-81^ restricted M134 TCR (PDB ID: 6V1A) are shown in *sticks*. P4 residue of cit-epitopes for (*A*) TNC^1014,1016cit^, (*B*) vimentin^64cit59-71^, (*C*) α-enolase^15cit10-22^, and (*D*) fibrinogen β^74cit69-81^ are colored in *orange*, *pink*, *light purple*, and *teal*, respectively. The SE residues of HLA-DRB1^∗^04:01 β-chain are presented in *yellow sticks*, whereas the CDR1α, 2α, 3α, and 3β loops are colored in *cyan*, *violet*, *light green* and *dark green*, respectively. *Black dashes* represent H-bond within 3.5 Å distance, *beige dashes* correspond to VdW interaction within 4 Å distance. *E*, overlaid SE residues of above mentioned four different cit-epitopes ternary complexes and P4-Asn/Cit. All amino acids are indicated in single-letter abbreviations. Citrulline denotes as Cit.

Given that the TNC^1014,1016cit^ peptide contains P4-Asn instead of P4-Cit (26, 27), we then analyzed the SE (^70^QKRAA^74^) interaction with P4-Asn of TNC^1014,1016cit^ peptide and PB TCR to understand the role of HLA-DRB1^∗^04:01 SE in PB TCR recognition (Fig. 6*A*). Here, we showed that the P4-Asn of TNC^1014,1016cit^ peptide is anchored with Lys^71^ of SE, and multiple contacts are formed between other SE residues (Gln^70^, Arg^72^, and Ala^73^) and CD2α (Tyr^57^ and Lys^58^) and CDR3β (Val^109^, Pro^110^, and Pro^111^) (Fig. 6*A*). A constant pattern of dual-recognition between Lys^71^ of SE and P4-Cit/P4-Asn, as well as Gln^70^ of SE and TCR CDR loops, was observed in this study, alongside three previously reported citrullinated epitopes, including vimentin^64cit59-71^, a-enolase^15cit10-22^, and fibrinogen β^74cit69-81^, albeit with distinct CDR loops or residues involved, suggesting this pattern is a hallmark of SE-peptide-TCR recognition (Fig. 6, *A*–*D*). Superposition of TCR-pHLA complexes of TNC^1014,1016cit^, vimentin^64cit59-71^, a-enolase^15cit10-22^, and fibrinogen β^74cit69-81^ at the SE region revealed a highly similar pattern between P4-Cit/P4-Asn and SE residues, with certain flexibility arises from Gln^70^ and Lys^71^ due to rearrangement of the alpha helix of the HLA-DRB1^∗^04:01 β-chain (Fig. 6*E*). Accordingly, the HLA-DRB1^∗^04:01 SE plays a consistent role in shaping PB TCR recognition of TNC^1014,1016cit^ despite asparagine at position P4.

### Presentation of TNC^1014,1016cit^ peptide by other SE ^+^ HLA-DRB1 allomorphs

**Figure 7:**
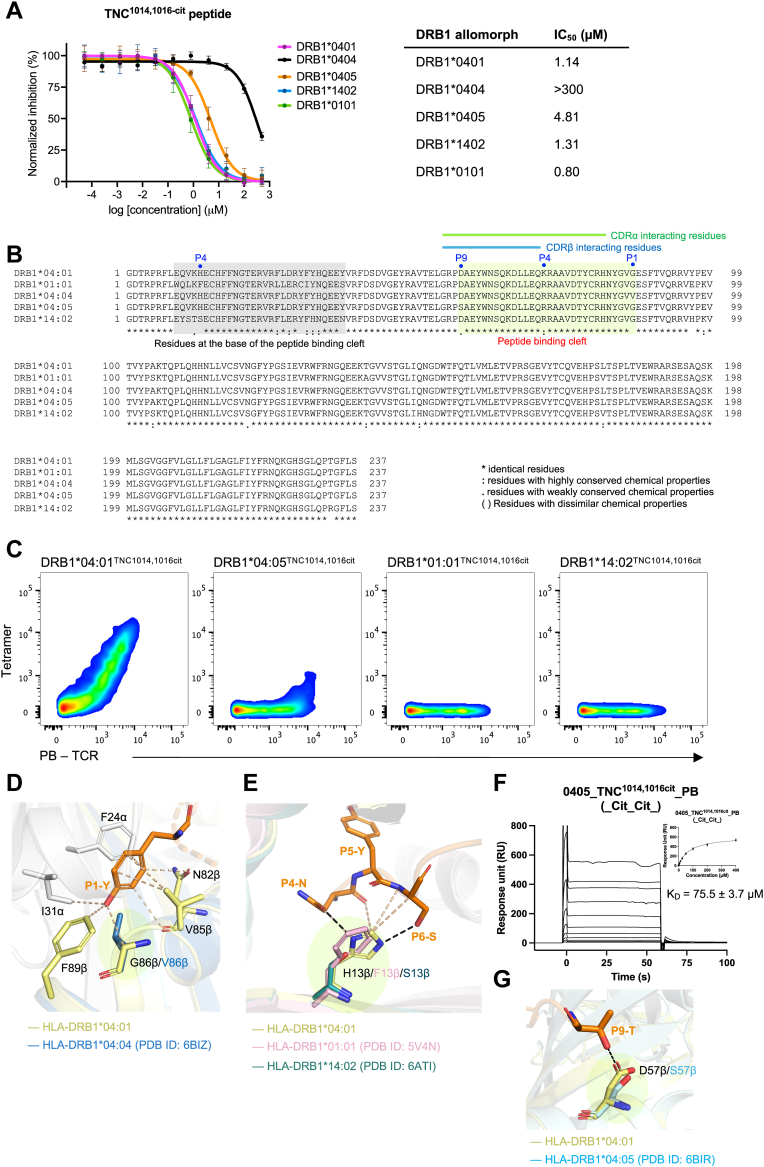
**PB TCR cross-reactivity towards other SE^+^ HLA-DRB1 allomorphs presenting TNC^1014,1016cit^**. *A*, titration curves of competitive binding of TNC^1014,1016cit^ peptide to DRB1^∗^04:01, ^∗^01:01, ^∗^04:04, ^∗^04:05, and ^∗^14:02 allomorphs. Each data point represents normalized relative binding (in percentage) for two independent experiments in *triplicate*, and the binding affinity at 50% inhibition of total binding was calculated as IC_50_ (μM) and the error bars correspond to ± s.e.m. *B*, multiple sequence alignment for *HLA-DRB1^∗^04*:*01*, *^∗^01*:*01*, *^∗^04*:*04*, *^∗^04*:*05*, and *^∗^14*:*02* alleles. Residues at the peptide binding cleft and form the base of the peptide binding cleft are highlighted in *light green* and *grey*, respectively. The conservation of the residues are denoted as ‘∗’ identical, ‘:’ highly conserved, and ‘.’ low similarity, and ‘space’ distinct. *C*, *in vitro* PB TCR expression and tetramer staining analysis for TNC^1014,1016cit^ peptide presented by HLA-DRB1^∗^04:01, ^∗^04:05, ^∗^01:01, and ^∗^14:02 allomorphs. Corresponding gating strategy is shown in Fig.S1. *D*, superposed HLA-DRB1^∗^04:01 (*yellow*) and ^∗^04:04 (*blue*) at P1-Tyr of TNC^1014,1016cit^ peptide. *E*, overlaid polymorphism at residue 13 of HLA-DRB1^∗^04:01 (*yellow*), ^∗^01:01 (*light pink*), and ^∗^14:02 (*teal*) and impact of interactions with P4-P6 of TNC^1014,1016cit^ peptide. *F*, binding affinity of PB TCR for HLA-DRB1^∗^04:05^TNC1014,1016cit^. HLA-DRB1^∗^04:01^CLIP^ was immobilized in the reference flow cell to control the non-specific binding. PB TCR equilibrium affinity constants (K_D_) value was determined from three independent experiments in *duplicate*. For each concentration, the point represents the mean value, and the error bar corresponds to ± s.e.m. *G*, the D57S polymorphism at the P9 binding pocket of HLA-DRB1^∗^04:01 and ^∗^04:05 (*pale cyan*) and the interaction with P9–Thr of TNC^1014,1016cit^. *Black dashes* represent H-bond within 3.5 Å, *beige dashes* correspond to VdW interaction within 4 Å. All amino acids are indicated in single-letter abbreviations.

Considering the high homology between SE^+^ HLA-DRB1 allomorphs, we next investigated if the TNC^1014,1016cit^ peptide can be presented by other SE ^+^ HLA-DRB1 allomorphs and impacted in cross-reactivity to PB TCR. We performed a peptide competition assay using the fluorescence polarization technique to measure the relative binding strength of TNC^1014,1016cit^ peptide for other HLA-DRB1 allomorphs (Fig. 7*A*). HA peptide that binds relatively strong to all HLA-DRB1 allomorphs, albeit weaker binding to DRB1^∗^04:04 was used as a control peptide (Fig. S4). As expected, HLA-DRB1^∗^01:01, ∗04:01, and ^∗^14:02 exhibited comparable strong binding to TNC^1014,1016cit^ peptide, with a IC_50_ of 0.8 μM, 1.1 μM, and 1.3 μM, respectively (Fig. 7*A*). Moreover, HLA-DRB1^∗^04:05 revealed more than fourfold weaker affinity when compared to HLA-DRB1^∗^01:01, ^∗^04:01, and ^∗^14:02. The relative binding strength of HLA-DRB1^∗^01:01, ^∗^04:01 and ^∗^14:02 fell within the “strong binding” range as previously established HLA-DRB1^∗^04:01 and cit-epitopes including cit-fibrinogen and cit-vimentin (14). In contrast, HLA-DRB1^∗^04:04 allomorph had limited binding to the TNC^1014,1016cit^ peptide, with a IC_50_ of over 300 μM and did not reach 100% inhibition (Fig. 7*A*). The sequence alignment of these SE^+^ HLA-DRB1 allomorphs at the peptide binding cleft revealed high sequence identity of the anchoring residues at the P1, P4, P6, and P9 pockets for *HLA-DRB1^∗^04*:*05*, *^∗^01*:*01*, and *^∗^14*:*02* alleles (Fig. 7*B*), consistent with established MHC II binding motif (34). Superposed crystal structures of HLA-DRB1^∗^04:01^TNC1014,1016cit^ and HLA-DRB1^∗^04:04 revealed that the HLA-DRB1^∗^04:04 allomorph did not bind the TNC^1014,1016cit^ peptide due to the hydrophobic Val^86^ in P1, which might inhibit the bulky aromatic tyrosine residue being accommodated in the P1 pocket due to steric clashes (Fig. 7, *B* and *D*).

### Cross-reactivity of PB TCR toward HLA-DRB1^∗^04:05 presenting TNC^1014,1016cit^ peptide

Next, we characterized the PB TCR cross-reactivity to other SE^+^ HLA-DRB1 allomorphs by transient expression and staining of PB TCR transfectants with individual DRB1^∗^04:05, ^∗^01:01, and ^∗^14:02 tetramers loaded with TNC^1014,1016cit^ peptide. Despite the observation that TNC^1014,1016cit^ peptide can bind to these three HLA-DRB1 allomorphs, PB TCR showed only some cross-reactivity to the HLA-DRB1^∗^04:05 allomorph presenting TNC^1014,1016cit^ peptide (Fig. 7*C*). This result suggests the polymorphism within the HLA-DRB1 allomorphs plays a role in TCR recognition. Structural alignment of HLA-DRB1^∗^04:01, ^∗^01:01, and ^∗^14:02 revealed a deviation at residue 13, which is located on the β-sheet floor with their side chains oriented into the peptide-binding groove. This feature is important for P4 and P6 anchoring. The substitution of His^13^ in HLA-DRB1^∗^04:01 to either Phe^13^ (DRB1^∗^01:01) or Ser^13^ (DRB1^∗^14:02), likely affects the rearrangement of the C-terminal region of the TNC^1014,1016cit^ peptide to avoid clashes or to improve binding to the base of the peptide-binding groove. This substitution will thus influence TCR recognition (Fig. 7, *B* and *E*).

Subsequent SPR analyses of PB TCR affinity for HLA-DRB1^∗^04:05^TNC1014,1016cit^ revealed a K_D_ of 75.5 μM, indicating the potential cross-reactivity of PB TCR to the DRB1^∗^04:05 allomorph (15) (Fig. 7*F*). The approximately threefold reduced in affinity of PB TCR towards HLA-DRB1^∗^04:05 ^TNC1014,1016cit^, as compared to HLA-DRB1^∗^04:01^TNC1014,1016cit^ was likely due to the polymorphisms embedded in the peptide binding cleft, which are located at the P9 and P4 pocket, where Asp^57^ and Lys^71^ in HLA-DRB1^∗^04:01 are substituted by Ser^57^ and Arg^71^ in DRB1^∗^04:05 allele, respectively (Fig. 7*B*). The Ser^57^ substitution in HLA-DRB1^∗^04:05 causes the loss of contact with P9-Thr of TNC^1014,1016cit^ peptide, likely affects the P9 anchoring, which is implicated in PB TCR docking (Fig. 7*G*). This is consistent with our FP assay result of reduced binding strength (IC_50_) for DRB1^∗^04:05^TNC1014,1016cit^ as compared to DRB1^∗^04:01^TNC1014,1016cit^. Collectively, the PB TCR restricted to the HLA-DRB1^∗^04:01, the strongest genetic RA risk factor in Europeans, can cross-react to highly conserved and susceptible RA allomorphs, namely, HLA-DRB1^∗^04:05 in Asians, albeit with weaker affinity.

## Discussion

Post-translational modification (PTM) of peptide antigens can confer the ability to bind to MHC II and have been implicated in immune disorders, as exemplified in citrullination in RA (14), deamidation of glutamine in coeliac disease (35), and peptide trans-splicing in type I diabetes (36, 37). In the context of RA, we have previously described the molecular basis for the specific TCR recognition of cit-fibrinogen (26), cit-vimentin (27), and cit-α-enolase (27) epitopes. We showed that citrullination at position P4 is not only critical in conferring the ability to occupy the P4 pocket of the SE in the HLA-DRB1^∗^04:01 but also has direct contact in TCR recognition (26, 27). In contrast, an additional citrullination at P2 of fibrinogen β^72,74cit69-81^ peptide had weaker binding to fibrinogen β^74cit69-81^-restricted TCRs, resulting in an altered TCR repertoire in immunized mice (26). In the present study, we focus on cit-tenascin-C peptide (TNC^1014,1016cit^), which contains two citrullination sites at positions P-1 and P2 beyond the P4-SE anchor, to further understand the impact of citrullination in TCR recognition. The crystal structure of PB TCR-HLA-DRB1^∗^04:01^TNC1014,1016cit^ revealed that while P5-Tyr is a distinct feature for TNC^1013-24^ peptide, citrullines at P-1 and P2 are the key determinants for PB TCR recognition. The synergistic effect of P-1 and P2 citrullines of TNC^1014,1016cit^ peptide confers high-binding affinity to PB TCR, suggesting the complementary role of multi-citrullination in TCR recognition. This is consistent with the SPR result, where single citrullination at the P-1 or P2 position of TNC^1013-24^ peptide either led to reduced affinity or completely abolished the interaction with PB TCR, respectively.

The lack of cross-reactivity observed for PB TCR towards other cit-RA autoantigens, including fibrinogen β^74cit69-81^, vimentin^64cit59-71^, and α-enolase^15cit10-22^, highlighted the strict specificity of PB TCR in TNC^1014,1016cit^ recognition, consistent with previously reported analysis (26, 27). Despite a highly conserved peptide binding register at P1, P4, and P6 across four different peptides, the distinct characteristics at positions P2 and P5 of cit-epitopes will discriminate against another cit-epitope restricted TCRs. For instance, in P5, the bulky aromatic feature of P5-Tyr in the TNC^1014,1016cit^ peptide, in comparison to small polar residues of P5-Ser of vimentin^64cit59-71^, P5-Pro of fibrinogen β^74cit69-81^, and P5-Gly of the α-enolase^15cit10-22^ peptide. In P2, there is a neutral P2-Cit in TNC^1014,1016cit^ peptide, as opposed to small residue P2-Ala, positively charged P2-Arg, and negatively charged P2-Asp in vimentin^64^^c^^it59-71^, fibrinogen β^74cit69-81^, and α-enolase^15cit10-22^ epitopes, respectively. These features indicate that the likelihood of cross-reactivity between cit-epitope-restricted TCRs is unlikely. Although TCR cross-reactivity has been reported in other autoimmune disorders such as coeliac disease (38) and Type 1 diabetes (37); nevertheless, these antigens involved shared high sequence homology. In contrast, the immunodominant epitopes recognized by TCRs in RA are peptide antigens with very diverse and distinct features that originate from different tissues. It is therefore not surprising that little or no cross-reactivity was observed between different cit-epitopes. Nevertheless, we observed constant duality recognition patterns of SE-TNC^1014,1016cit^ peptide and PB TCR interaction, consistent with our previously described fibrinogen β^74cit69-81^, vimentin^64cit59-71^, and α-enolase^15cit10-22^ TCR-pMHC II complexes. This was the case with either a neutral asparagine or a neutral citrulline at P4, thereby affirming the role of SE^+^-HLA in TCR recognition (26, 27).

Moreover, we demonstrated the capability of the TRAV35^+^/TRBV10-2^+^ PB TCR to cross-react with the highly homologous HLA-DRB1^∗^04:05 allomorph, albeit with weaker affinity. The distinct polymorphism underlying the peptide-binding groove is located at P9 pocket, with a Ser^57^ residue in HLA-DRB1^∗^04:05, as opposed to Asp^57^ in HLA-DRB1^∗^04:01. This residue at position 57 was reported to have a susceptibility effect which accounts for the detrimental association between the SE and joint destruction in Japanese patients with ACPA-positive RA (16). Furthermore, although the PB TCR did not recognize citrullinated TNC^1014,1016cit^ peptide presented by HLA-DRB1^∗^01:01 or ^∗^14:02, there is certainly a potential that additional TCRs may have the capacity to recognize this peptide also in the context of those alleles. The deviation at residue 13 of HLA-DRB1^∗^04:01, ^∗^01:01, and ^∗^14:02 that affects the binding preferences of PB TCR, likely contributes to odds ratio of RA pathogenesis, consistent with reported study of residue 13 is as important as the SE in associated with seropositive RA (15). Overall, our study has provided a structural insight into how citrullination shapes specific CD4^+^ T cell recognition in RA.

## Experimental procedures

### Peptide

Peptides including TNC^1014,1016cit^ (^1013^DcitYcitLNYSLPTG^1024^, where cit represents citrulline residue), native TNC^1013-24^ (^1013^DRYRLNYSLPTG^1024^), TNC^1014cit^ (^1013^DcitYRLNYSLPTG^1024^), TNC^1016cit^ (^1013^DRYcitLNYSLPTG^1024^), Vimentin^64cit59-71^ (^59^GVYATcitSSAVRLR^71^), α-enolase^15cit10-22V20G^ (^10^EIFDScitGNPTGEV^22^), Fibrinogen β^74cit69-81^ (^69^GGYRAcitPAKAAAT^81^), and HA^306-318^ (^306^PKYVKQNTLKLAT^318^) were synthesized by GL Biochem. The integrity of the peptides was verified by reverse-phase high-performance liquid chromatography and mass spectrometry.

### Protein expression and purification

The TCR α- and β-chains were designed and expressed as previously described (26, 39). In brief, the extracellular domains of TCR α- and β-chains were engineered with a disulfide linkage in the constant domains to stabilize the heterodimer. TCR α- and β-chains were then expressed independently as inclusion bodies in *Escherichia coli* BL21 (DE3) and, subsequently, refolded in a buffer containing 5 M Urea, 100 mM Tris pH 8.0, 0.4 M L-Arginine, 2 mM EDTA, 0.2 mM phenylmethylsulfonyl fluoride, 0.5 mM oxidized glutathione, and 5 mM reduced glutathione for 72 h at 4 °C with rapid stirring. The refolded samples were dialyzed with 10 mM Tris pH 8.0 and purified on a DEAE (Cytiva) anion exchange column, followed by size exclusion (HiLoad 16/600 Superdex 200pg column; Cytiva), hydrophobic interaction (HiTrap Phenyl HP column; Cytiva), and anion exchange (HiTrap TM Q HP column; Cytiva) chromatography.

The expression of HLA-DRB1^∗^04:01 was performed as described in previous article (27). Briefly, the extracellular domains of the α-chain and β-chain of HLA-DRB1^∗^04:01 (*HLA-DRA^∗^01*:*01* and *HLA-DRB1^∗^04*:*01*) were covalently linked to invariant chain (CLIP) peptide, and cloned into the lentiviral vectors, namely, pLV-EF1α-MCS-IRES-GFP and pLV-EF1α-MCS-IRES-RFP (Biosettia), respectively. The HLA-DRB1^∗^04:01 lentivirus was produced by co-transfection of these vectors, along with viral packaging plasmids (pMD2.G, pMDLg/pRRE, pRSV-REV; Addgene), in the human embryonic kidney (HEK) 293 T cells. The HLA-DRB1^∗^04:01 lentivirus was harvested and transduced into glycosylation deficiency HEK293S (GnTi^-^) (CRL-3022, ATCC) cells and subsequently sorted by single-cell FACS (Becton Dickinson) to generate a cell line that stably expresses HLA-DRB1^∗^04:01. To produce HLA-DRB1^∗^04:01 protein, stably expressed clones were cultured in Expi293 Expression Medium (serum free media; Gibco, Thermo Fisher Scientific) in shaking incubator at 37 °C in 5% CO_2_. HLA-DRB1^∗^04:01 protein was then harvested and purified as previous described (13). Briefly, the supernatant containing soluble HLA-DRB1^∗^04:01 protein was concentrated and dialysed to 10 mM Tris pH 8.0 and 150 mM NaCl using tangential flow filtration (TFF) on a Cogent M1 TFF system (Merck Millipore), followed by subsequent purification *via* immobilized metal ion affinity (Nickel-Sepharose 6 Fast Flow; Cytiva), and size exclusion (Superdex 200, 16/600; Cytiva) chromatography.

The construct design and expression of other HLA-DRB1 proteins (*HLA-DRA1^∗^01*:*01*, *^∗^04*:*04*, *^∗^04*:*05*, *^∗^01*:*01* or *^∗^14*:*02*) were as previously described (14). The C-terminus of the DRB1 α-chain had a Fos leucine zipper, and the β-chain had a Jun leucine zipper, followed by a BirA biotin ligase biotinylation recognition sequence and a polyhistidine tag. The N-terminus of the β-chain was covalently linked to a factor Xa-cleavable Strep-tag invariant chain (CLIP) peptide. The extracellular domains of α- and β-chains were independently cloned into the pHLsec vector, transfected using polyethyleneimine (PEI) (BioScientific) at a ratio of 1:3 of DNA to PEI. The transfected cells were incubated at 37 °C with 5% CO_2_ in a 120-rpm shaker incubator for a week. The soluble recombinant HLA-DRB1 proteins were then purified from the cell culture supernatant as stated above. Purified monomeric peptide-HLA-DRB1 was biotinylated using biotin protein ligase (BirA) in buffer containing 0.05 M bicine pH 8.3, 0.01 mM ATP, 0.01 mM MgOAc, 50 μM d-biotin, and 2.5 μg BirA. BirA was made according to protocols outlined in O'Callaghan C *et al*. (40).

### Peptide loading of HLA-DRB1

The HLA-DRB1 proteins presenting CLIP peptide were digested with Factor Xa (New England Biolabs) to cleave the covalently linked Strep-CLIP in TBS150 buffer (10 mM Tris pH 8.0, 150 mM NaCl) containing 2 mM CaCl_2_ for 6 h at room temperature. 5 mM EDTA was used to stop the enzymatic reaction. The cleaved HLA-DRB1 was subsequently loaded with 20 M excess of peptide in 50 mM trisodium citrate pH 5.4 in the presence of HLA-DM at a molar ratio of 5:1 and incubated for 72 h at 37 °C. The peptide-loaded HLA-DRB1 was passed through a Strep-Tactin Sepharose (IBA) column to remove the partially digested or unloaded HLA-DRB1-Strep-CLIP.

### *In vitro* TCR expression and tetramer staining

HEK 293T cells (ATCC, #CRL-3216) were plated at 3.5 x 10^5^ cells/well of a six well plate in 3 ml RF10 media containing RPMI-1640, 10% fetal bovine serum (FBS, Sigma), glutamax (Gibco, #35050061), Non-essential amino acid (Gibco, #11140050), HEPES (Gibco, #15630130), sodium pyruvate (Gibco, #11360070), penicillin-streptomycin (Gibco, #15070063), 50 μM 2-mercaptoethanol (Merck), for 24h at 37 °C, 5% CO_2_. 420 ng of individual lentiviral vector pLV-EF1α-MCS-IRES-GFP encoding TCR α-chain and pLV-EF1α-MCS-IRES-RFP (Biosettia) encoding TCR β-chain were transiently expressed together with the pLV encoding CD3γδεζ subunits in HEK 293T cells using FuGene 6 HD (Promega, #E2691). On the following day, transfected HEK 293T cells were detached and repeatedly washed with FACS buffer (PBS + 2% FBS) by centrifugation at 350*g* for 5 min, prior being labelled with 0.5 μg of individual peptide-loaded HLA-DRB1 tetramer for 1 h in dark at room temperature. Cells were then stained with 1:100 diluted BUV395 mouse anti-human CD3 antibody (clone UCHT1, BD Biosciences) for 1 h in dark at 4 °C, washed three times with FACS buffer, followed by live/dead cell staining with 1:10,000 diluted 4′,6-diamidino-2-phenylindole (DAPI; BD Biosciences) viability stain for 15 min before being analyzed on a BD LSRFortessa X-20 with FACSDiva 8.0.1 software (BD Immunocytometry Systems). Three independent experiments were conducted for all tetramer staining analysis. Collected data were analyzed using FlowJo v10.9.0 (Flowjo).

### Surface plasmon resonance

The affinity measurements were performed using surface plasmon resonance on a Biacore T200 instrument (Cytiva). Approximately 3000 response units (RU) of biotinylated peptide-loaded HLA-DRB1 were immobilized on a streptavidin (SA) sensor chip (Cytiva). HLA-DRB1^∗^04:01^CLIP^ was immobilized in the reference flow cell and acted as negative control. Serial dilutions of TCRs were passed over the flow cell's surface at the rate of 10 μl/min in 20 mM HEPES pH 7.5, 150 mM NaCl, 1 mM EDTA, and 0.005% v/v surfactant P20 (Cytiva). Three independent experiments in duplicate were performed for PB TCR, and two independent experiments in duplicate were performed for PB TCR mutants. Collected data were analyzed on Prism 10 (GraphPad Software, version 10.2.0) using a one-site specific binding model and plotted as sensorgrams and equilibrium response curves. M134 TCR (26) and A03 TCR (27) were used as negative control TCRs.

### T cell stimulation assay

The PB TCR α- and β-chains were cloned into pLV-EF1α-MCS-IRES-GFP and pLV-EF1α-MCS-IRES-RFP, respectively, and subsequently transduced into the SKW3 T cell line (TCR deficient; German Collection of Microorganisms and Cell Cultures) for stable expression using the lentiviral transduction system as previously described (41). PB TCR-transduced SKW3 T cells were cultured in RF10 media at 37 °C in 5% CO_2_. Briefly, approximately 0.1 x 10^6^ BLCL 9031 cells (*HLA-DRA1^∗^01*:*01*, *HLA-DRB1^∗^04*:*01*; sourced from The International Histocompatibility Working Group (IHWG) Cell and DNA Bank) acting as antigen presenting cells, were incubated with serial dilutions of TNC^1014,1016cit^ peptide (5:1 dilution starting at 5 μg/ml), 50 μg of α-enolase^15cit10-22^ peptide (negative control), or 35 μg/ml of anti-HLA-DR monoclonal antibody (clone LB3.1, blocking antibody; negative control) in 96-well round-bottom plate (Corning) for 4 h at 37 °C, 5% CO_2_. Subsequently, 1 x 10^5^ PB TCR transduced SKW3 T-cells, RA2.7 TCR transduced SKW3 T-cells (control) or untransduced SKW3 parental cells were added to the wells accordingly and incubated overnight at 37 °C, 5% CO_2_. On the following day, the cells were then washed twice with FACS buffer, then stained with a mixture of 1:100 diluted BUV395 mouse anti-human CD3 (clone UCHT1, BD Biosciences), and APC mouse anti-human CD69 (clone FN50, BD Biosciences) for 1 h at 4 °C in dark. Cells were then washed 6 times with FACS buffer to remove excess antibodies, followed by live/dead staining with DAPI (BD Biosciences) at 1:10,000 ratio for 15 min. Subsequently, cells were analyzed *via* flow cytometry using FACSDiva 8.0.1 software on the BD LSRFortessa X-20 (BD Immunocytometry System). Three independent experiments were conducted, and all samples were performed in duplicate. Collected data were analyzed using FlowJo v10.9.0 and plotted with Prism 10 (GraphPad Software, version 10.2.0). One-way ANOVA multiple comparison with Dunnett’s multiple comparison testing was used to determine the statistical significance between the MFI values of unstimulated SKW3 PB T-cells *versus* the peptide-stimulated SKW3 PB T-cells.

### Crystallization, data collection, and processing

For crystallization, the monomeric TNC^1014,1016cit^ loaded HLA-DRB1^∗^04:01 was subjected to HRV 3C protease to remove C-terminal Fos/Jun leucine zipper tagging. For the ternary complex, HLA-DRB1^∗^04:01^TNC1014,1016cit^ was mixed with PB TCR at 1:1 M ratio and incubated for 6 h at room temperature. Proteins were concentrated up to 10 mg/ml and undertook high-throughput crystallization screening at the Monash Molecular Crystallization Platform (MMCP) using an automated robotic NT8 system. The HLA-DRB1^∗^04:01^TNC1014,1016cit^ binary complex was crystallized in reservoir solution containing 0.1 M Bis-Tris pH 5.5, 0.2 M NaCl and 29% w/v PEG3350; the PB TCR – HLA-DRB1^∗^04:01^TNC1014,1016cit^ ternary complex was crystallized in 0.1 M Tris pH 7.5, 0.3 M NaCl, 0.05 M Glutamic Acid, 0.05 M Arginine and 20% w/v PEG3350; the apo PB TCR was crystallized in 0.1 M Na Acetate pH 7.8 and 8% w/v PEG 4K. Single crystals were treated with mother liquor containing cryoprotectant (15–25% glycerol or ethylene glycol) prior to flash freezing in liquid nitrogen. Diffraction data were collected at the Australian Synchrotron’s MX2 beamline, auto-processed and scaled with XDS and CCP4 Software Suite version 8.0.

### Structure determination, refinement, and validation

Crystal structures of PB TCR-HLA-DRB1^∗^04:01^TNC1014,1016cit^ ternary complex, PB TCR apo form, and HLA-DRB1^∗^04:01^TNC1014,1016cit^ binary were solved by molecular replacement in PHASER (CCP4 Software Suite, version 8.0) using a separate search model for HLA-DRB1^∗^04:01 and TCR (PDB ID: 6V1A) (26). Multiple rounds of model building in Coot (42) and automated refinement using Phenix.refine (PHENIX) (43). The quality of the structures was validated at the Protein Data Bank (PDB) validation and deposition server. The PB TCR structure was numbered according to the IMGT unique numbering system (44). Data processing and refinement statistics were summarized in Table 1. Ramachandran statistics of final models revealed ∼ 95%-97% of residues were in favored regions, with no outlier residue. Buried surface area and TCR-pHLA contact analyses were determined using the program Areaimol and Contact in CCP4 Program Suite, respectively. PyMOL (version 2.5.2) was used to generate all structural figures.

### Fluorescence polarization assay

The relative binding strength of TNC^1014,1016cit^ peptide for HLA-DRB1^∗^04:01, ^∗^01:01, ^∗^04:04, ^∗^04:05 and ^∗^14:02 was determined through the fluorescence polarization assay, as described previously (14, 45). In brief, serial dilution of peptide, starting from 500 μM was incubated in competition with 20 nM TAMRA-HA fluorescent labeling peptide, to bind with 100 nM HLA-DRB1 protein in the presence of 20 nM HLA-DM, in the buffer comprising of 100 mM trisodium citrate pH 5.4, 50 mM NaCl and 5 mM EDTA. The fluorescent polarization was measured by PHERAstar microplate reader (BMG LABTECH) after 24 h, 48 h and 72 h incubation at 37 °C. The peptide binding curves were plotted by non-linear regression in Prism 10 (GraphPad Software, version 10.2.0) using a sigmoidal dose-response curve. IC_50_ values were calculated as the peptide concentration required for 50% inhibition of TAMRA-HA fluorescent labeling peptide binding to HLA-DRB1 protein. All data were derived from two independent experiments in triplicate.

## Data availability

The X-ray crystal structures were deposited in the Protein Data Bank (PDB) with the following accession codes: DRB1^∗^04:01^TNC1014, 1016cit^, 9NIH; PB TCR-DRB1^∗^04:01^TNC1014,1016cit^, 9NIG; PB TCR, 9NII.

## Supporting information

This article contains supporting information.

## Conflict of interests

The authors declare that they have no conflicts of interest with the contents of this article.
