# Supplementary material for: The molecular basis of T cell receptor recognition of citrullinated tenascin-C presented by HLA-DR4
Source: J Biol Chem. 2025 Jun 2;301(7):110326. doi: 10.1016/j.jbc.2025.110326 (PMC12269843; doi:10.1016/j.jbc.2025.110326)
Supplement: Supplementary Materials [file mmc1.pdf]

Supporting information

The molecular basis of T cell receptor recognition of citrullinated tenascin-C presented by HLA-DR4

Hien Thy Dao[1], Tiing Jen Loh[1], Ravi K. Sharma[2,3], Lars Klareskog[2], Vivianne Malmström[2], Hugh H. Reid[1], Jamie Rossjohn[1,4*] & Jia Jia Lim[1*]

[1]*Infection and Immunity Program and Department of Biochemistry and Molecular Biology, Biomedicine Discovery Institute, Monash University, Clayton, Australia*

[2]*Division of Rheumatology, Department of Medicine, Karolinska Institutet, Karolinska University Hospital, Stockholm, Sweden; Center for Molecular Medicine, Karolinska Institutet, Solna, Sweden.*

[3]*Department of Clinical Immunology and Rheumatology, All India Institute of Medical Sciences, Bilaspur (H.P), India.*

[4]*Institute of Infection and Immunity, Cardiff University School of Medicine, Heath Park, Cardiff, United Kingdom.*

* Joint senior and corresponding authors: Jiajia.lim@monash.edu, Jamie.Rossjohn@monash.edu

Supporting Figures 1-4

Supporting Tables 1-3

Gating strategies

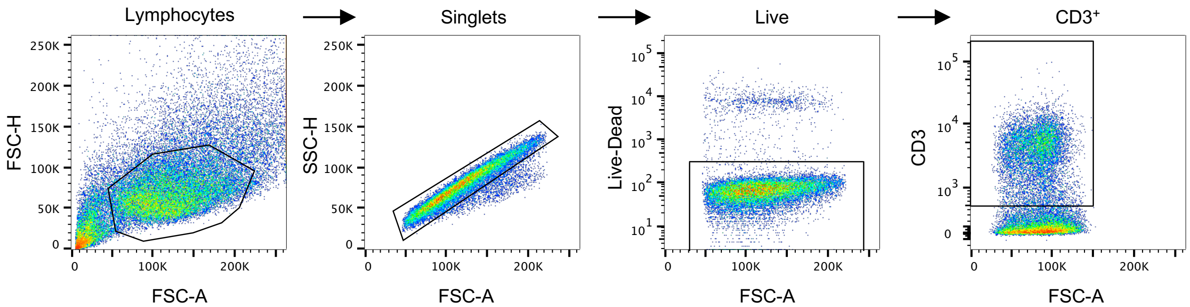

Supporting Figure 1. Gating strategy for the tetramer staining analysis. HEK 293T cells transiently co-transfected with TCR and CD3$\gamma\delta\varepsilon\zeta$ were gated based on size to select single lymphocytes, followed by viability gate (DAPI) for live cells and finally $CD3^{+}$ cells.

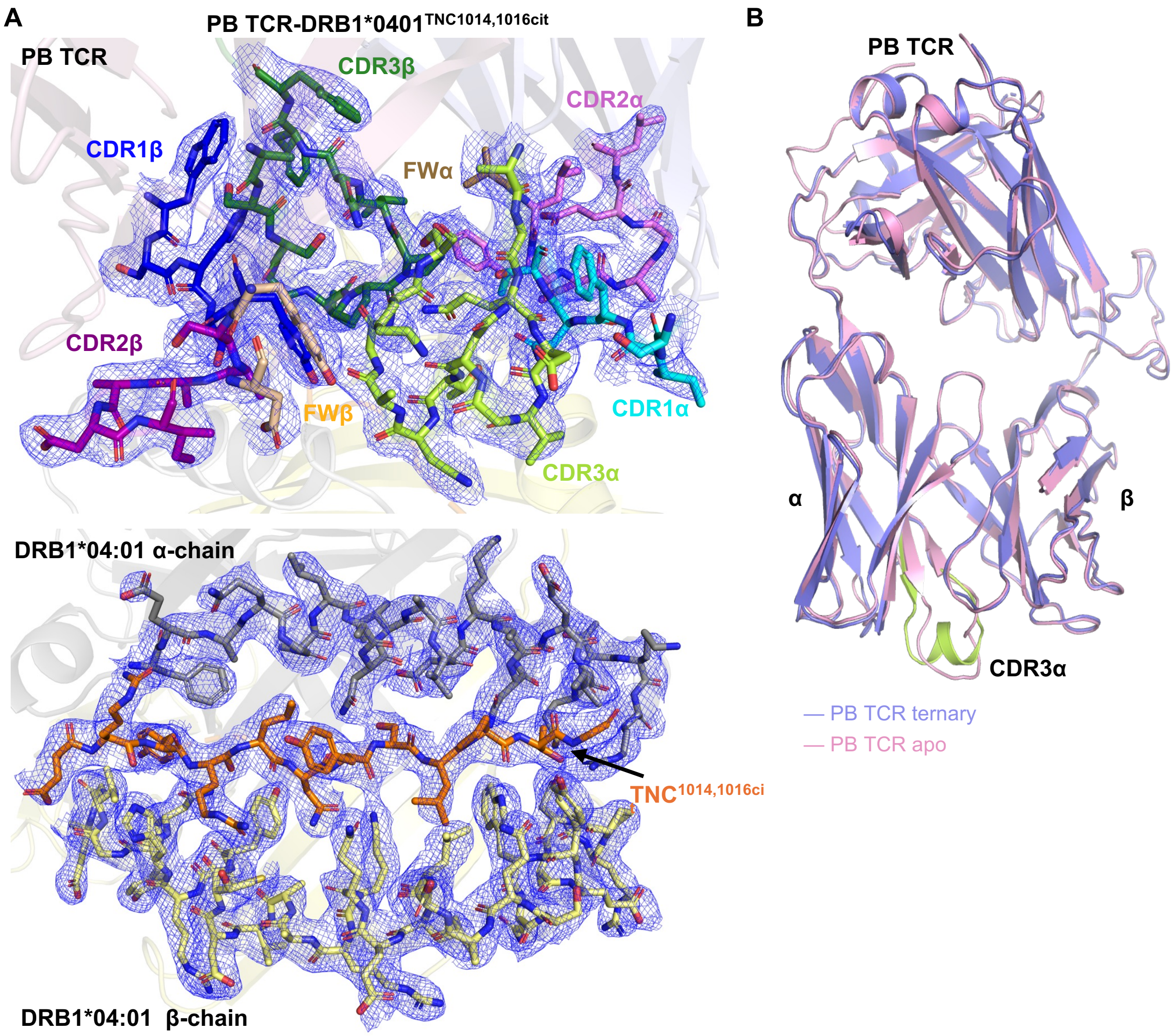

Supporting Figure 2. Electron density map for the interface residues of PB TCR and HLA-DRB1*04:01^{TNC1014,1016cit}, and superposed structure of PB TCR from PB TCR-HLA-DRB1*04:01^{TNC1014,1016cit} ternary complex and unliganded apo PB TCR. (a) The $2mF_o - DF_c$ electron density map for the interface residues of (Top) PB TCR and (Bottom) HLA-DRB1*04:01^{TNC1014,1016cit} were shown in blue and contoured at 1σ. The CRD loop 1α, 2α and 3α are highlighted in cyan, violet and light green, whereas 1β, 2β and 3β are colored in blue, purple and dark green, respectively. The FWα residue is colored in sand and β residues are colored in beige. The HLA-DRB1*04:01 α- and β-chain are presented in grey and yellow, respectively, while TNC^{1014,1016cit} peptide are shown in orange. **(b)** Superimpose PB TCR structure from PB TCR-HLA-DRB1*04:01^{TNC1014,1016cit} ternary complex and unliganded apo PB TCR, displaying the different conformation of the CDR3α loop. PB TCR from ternary complex are presented as purple cartoon with the CDR3α loop highlighted in light green, whereas the unliganded apo PB TCR structure are shown in light pink cartoon.

PB TCR mutants - HLA-DRB1*04:01^{TNC1014,1016cit}

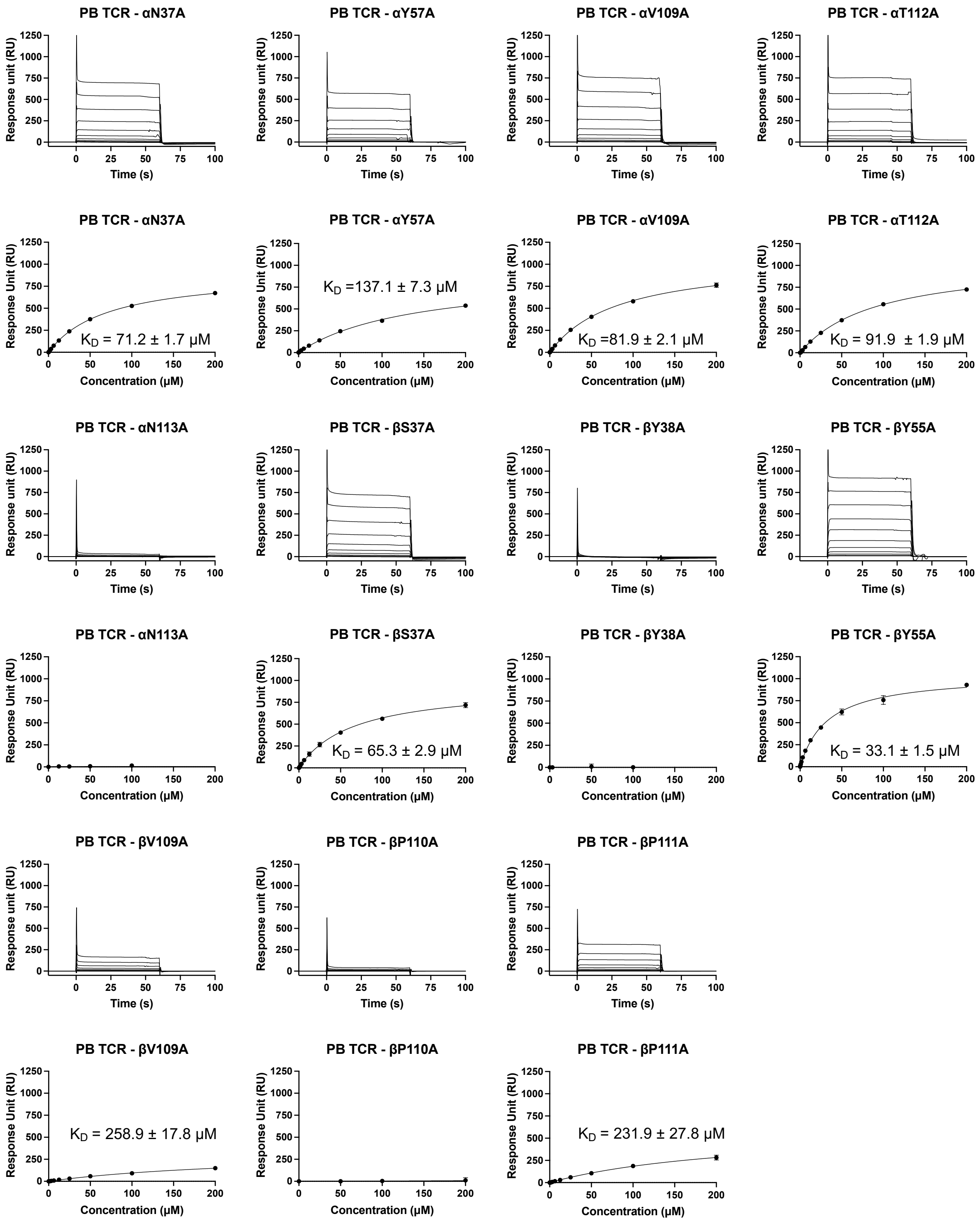

Supporting Figure 3. Affinity analysis of PB-TCR point mutants for HLA-DRB1*04:01^{TNC1014,1016cit}. The equilibrium affinity constants (K_D) were determined from two independent experiments in *duplicate* and a single ligand binding model was used to fit the curves. The maximal TCR concentration was 200 μM. To control for non-specific binding, HLA-DR4^{CLIP} was immobilized in the reference flow cell and used as baseline value. For each concentration, the points represent the mean value, and the error bars correspond to SD.

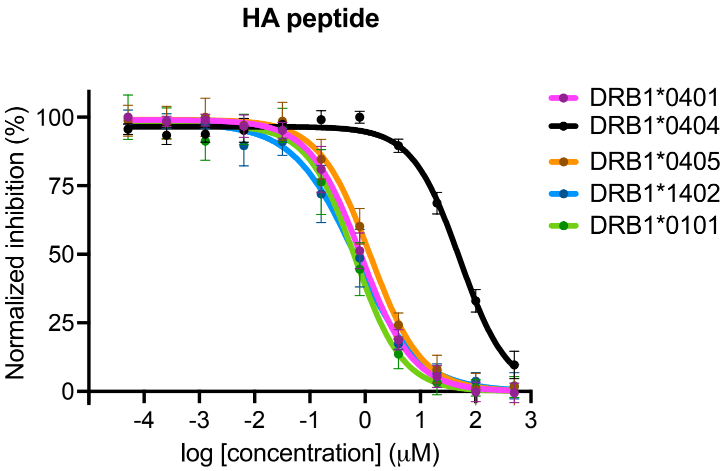

DRB1 allomorph	IC_{50} (μM)
DRB1*0401	0.85
DRB1*0404	51.6
DRB1*0405	1.24
DRB1*1402	0.68
DRB1*0101	0.66

Supporting Figure 4. Fluorescence polarization peptide competitive assay of SE⁺HLA-DRB1 allomorphs. Titration curves of competitive binding of control HA peptide to DRB1*04:01, *01:01, *04:04, *04:05, and *14:02 allomorphs. Each data point represents normalized relative binding (in percentage) for two independent experiments in *tripl*icate with the error bars correspond to SE, and the binding affinity at 50% inhibition of total binding was calculated as IC_{50} (μM).

Table S1. PB TCR-HLA-DRB1*04:01^{TNC1014,1016cit} complex statistics

	BSA (Å)	Vα (%)	Vβ (%)	Peptide (%)	1α (%)	2α (%)	3α (%)	Fwα (%)	1β (%)	2β (%)	3β (%)	Fwβ (%)
**PB TCR-HLA-DRB1*04:01^{TNC1014,1016cit}**	1912.3	62	38	33.4	4.4	21.1	32.8	3.7	7.6	9.6	17.1	3.7

Table S2. Contacts of PB TCR-HLA-DRB1*04:01^{TNC1014,1016cit}

TCR segment	TCR residues	HLA-DRB1*04:01	Type of bond
**CDR1α**	Asn-37	Thr-77β	VdW
**CDR2α**	Tyr-57 (OH)	Asp-66β (OD1), Glu-69β, Ala-73β, Gln-70β	H-bond, VdW
	Lys-58 (NZ)	Glu-69β (OE2), Arg-72β	SB, VdW
	Glu-64	Glu-69β	VdW
**CDR3α**	Val-109	His-81β	VdW
	Asn-113 (O)	Gln-57α (N), Gly-58α (N) Phe-54α, Glu-55α, Ala-56α	H-bond, VdW
	Ala-114	Gln-57α	VdW
**FWα**	Ala-55 (M)	Asp-66β	VdW
**CDR1β**	Ser-37	Ala-61α	VdW
	Tyr-38	Gly-58α, Ala-61α	VdW
**CDR2β**	Ala-57	Gln-57α, Ala-61α	VdW
	Ala-58	Ala-64α	VdW
	Ile-65	Leu-60α	VdW
**CDR3β**	Val-109	Leu-67β, Gln-70β	VdW
	Pro-110 (O)	Gln-70β (NE2)	H-bond, VdW
	Pro-111	Gln-70β	VdW
**FWβ**	Tyr-55 (OH)	Gln-57α (NE2)	H-bond, VdW
	Asp-67	Gln-57α	VdW

TCR segment	TCR residues	TNC^{1014,1016cit}	Type of bond
**CDR1α**	Asn-37 (OD1)	P2-Cit (N8)	H-bond, VdW
**CDR3α**	Gln-107 (NE2)	P5-Y (OH)	H-bond, VdW
	Val-109	P1-Cit, P1-Y, P2-Cit	VdW
	Gly-110	P1-Cit	VdW
	Thr-112 (O), (OG1)	P5-Y (OH), P2-Cit (N8), P3-L	H-bond, VdW
	Asn-113 (ND2), (OD1)	P-1-Cit (O7), P1-Y (O), P3-L (N), P2-Cit	H-bond, VdW
	Gly-115	P5-Y	VdW
**CDR1β**	Tyr-38	P5-Y	VdW
**CDR3β**	Val-109	P5-Y	VdW
	Pro-110	P5-Y	VdW

VdW: Van der Waals interaction (cut-off at 4 Å)

H-bond: hydrogen bond (cut-off at 3.5 Å)

SB: salt bridge (cut-off at 4.5 Å)

Table S3: K_D values and fold of affinity of PB TCR mutants

PB TCR mutants	K_D (μM)	Fold
αN37A	71.2 ± 1.7	2.8 ± 0.3
αY57A	137.1 ± 7.3	5.3 ± 0.3
αV109A	81.9 ± 2.1	3.2 ± 0.2
αT112A	91.9 ± 1.9	3.6 ± 0.2
αN113A	NB	NB
βS37A	65.3 ± 2.9	2.5 ± 0.3
βY38A	NB	NB
βY55A	33.1 ± 1.5	1.3 ± 0.01
βV109A	258.9 ± 17.8	10.1 ± 0.4
βP110A	NB	NB
βP111A	231.9 ± 27.8	9.0 ± 0.03

NB: No binding
